# Chronic myeloid leukemia incidence, survival and accessibility of tyrosine kinase inhibitors: a report from population-based Lithuanian haematological disease registry 2000–2013

**DOI:** 10.1186/s12885-016-2238-9

**Published:** 2016-03-08

**Authors:** Tumas Beinortas, Ilma Tavorienė, Tadas Žvirblis, Rolandas Gerbutavičius, Mindaugas Jurgutis, Laimonas Griškevičius

**Affiliations:** Clinical Medical School, University of Oxford, Oxford, UK; Centre for Evidence-Based Medicine, Clinics of Internal, Family Medicine and Oncology, Faculty of Medicine, Vilnius University, M. K. Ciurlionio str. 21, 03101 Vilnius, Lithuania; Hematology, Oncology and Transfusion Medicine Center, Vilnius University Hospital Santariskiu Klinikos, Santariskiu 2, 08661 Vilnius, Lithuania; Clinics of Oncology and Hematology, Hospital of Lithuanian University of Health Sciences Kauno Klinikos, Eivenių g. 2, 50009 Kaunas, Lithuania; Department of Oncology Haematology, Klaipeda Seamen Hospital, Liepojos 45, 92288 Klaipeda, Lithuania; Clinics of Internal, Family Medicine and Oncology, Faculty of Medicine, Vilnius University, M. K. Ciurlionio str. 21, 03101 Vilnius, Lithuania

**Keywords:** Chronic myeloid leukemia, Tyrosine kinase inhibitors, Survival, Lithuania, Drug penetrance, Drug availability, Europe

## Abstract

**Background:**

Currently available chronic myeloid leukaemia (CML) survival reports have originated from more affluent countries. Herein we report the entire country data on incidence and survival of CML, as well as penetrance of tyrosine kinase inhibitors (TKIs) in Lithuania.

**Methods:**

We analyzed all patients (*N* = 601) from the national haematological disease monitoring system who were diagnosed with CML between 2000 and 2013. Crude (CR) and age-standardized (weighted) (ASW(R)) incidence and mortality rates, as well as 1-, 5-, and 10-year relative survival rates (RSR) were calculated. Information on TKI penetration is also reported.

**Results:**

Throughout the entire 2000–2013 period the median age at diagnosis of CML patients was 62 years. The respective incidence and mortality CRs were 1.28 and 0.78, both characterized by decreasing trends over the observation period. A 5-year RSR increased from 0.33 [95 % CI, 0.27–0.40] in 2000–2004 to 0.55 [95 % CI, 0.47–0.63] in 2005–2009. However, the respective 5-year RSRs for patients aged 65–74 and ≥75 were only 0.33 [95 % CI, 0.24–0.42] and 0.18 [95 % CI 0.07–0.23] during the entire study period. TKI penetrance for CML patients grew from 1.5 % in 2000–2004 to 30.6 % in 2005–2009 and 69.1 % in 2010–2013. TKI penetrance was low in the older age groups (60 % for the 65–74 and 19 % for the ≥75 patient group, in 2010–2013).

**Conclusion:**

Relative CML survival in Lithuania steadily improved and paralleled the increase in TKI treatment availability. Patients above 64 years rarely received TKIs and their relative survival remained low throughout the observation period. The latency of TKI availability may have influenced the survival trends.

**Electronic supplementary material:**

The online version of this article (doi:10.1186/s12885-016-2238-9) contains supplementary material, which is available to authorized users.

## Background

The approval of Imatinib mesylate in 2001 [1] and thereafter emergence of second generation Bcr-Abl1 inhibitors have transformed Chronic Myeloid Leukaemia (CML) from deadly to readily treatable cancer [2, 3]. IFNα and chemotherapy were rapidly replaced by imatinib as a mainstay treatment of CML, after clinical trial data demonstrated the treatment effect that few cancer treatments have shown before [4–6].

Although in some patients tyrosine kinase inhibitors (TKIs) may cure CML, the discontinuation of imatinib treatment in prolonged molecular remission usually leads to the molecular relapse of the disease [7]. Therefore to acquire a survival benefit, most of CML patients need to stay on TKIs for the rest of their life. With ever expanding CML patient population the financial sustainability of expensive Bcr-Abl1 inhibitors has been debated even in economically well-established countries [8]. Current cost-effectiveness and cost-utility analyses are based on data from clinical trials, which delineate strict patient inclusion criteria and provide rigorously controlled treatment regimens at university hospitals [9, 10]. But in real life patients and treatment quality in different centers may be more variable, thus clinical trials may not always accurately reflect the treatment efficacy at a country-wide population level [11]. Population studies readily address these challenges and can accurately describe the incidence, prevalence and real-life survival of target disorder.

Currently available population CML survival data has largely originated from economically well-established countries [2, 3, 12–15]. However, some of CML survival population studies rely exclusively on regional registries linked to haematological specialty centers, rather than national cancer registries, and therefore are susceptible to referral and selection bias [3, 14]. Complete population reports minimize the risk of selection bias, but entire country population CML incidence and survival reports are currently accessible for Sweden and United Kingdom only [2, 12, 13].

The transitional nature of Lithuania’s economy has limited the availability of cancer medicines under patent: imatinib became partially available in 2005 and fully available only in 2011. In this study we report an unselected entire country population data on CML incidence, survival and TKI penetrance in Lithuania from 2000 to 2013. We also aim to compare the CML survival differences between countries due to differences in the availability of the innovative treatment.

## Methods

### Lithuanian HESS registry

Lithuania has a national haematological disease monitoring system (HESS), which collects data from 2000 and covers the entire country, with a population of 3 million. Patients with haematological malignancies are managed in 5 centres across the country and all physicians and pathologists are obliged to report all newly diagnosed CML cases to HESS registry. HESS contains data on age, sex, ICD-10 code, date of diagnosis, clinical symptoms, laboratory test, risk group, treatment, Ph and BCR-ABL status (both mandatory from 2010) of CML. Eastern Cooperative Oncology Group (ECOG) performance score and CML phase at presentation were collected from 2010. Through unique personal ID, HESS is also linked to the national death registry, which allows further validation of data. All Lithuanians are covered by national healthcare insurance and haematological diseases are treated in public healthcare system. Therefore underreporting to HESS registry is unlikely. All patients, who were diagnosed with CML (ICD-10 code 92.1) between January 1st 2000 and December 31st 2013, were entered into the study. There was no age restriction or other exclusion criteria. The study was conducted according to the declaration of Helsinki and was approved by the Lithuanian Bioethics Committee, which also waived the need for informed consent.

### Statistical methods

Descriptive statistics were used to analyze patients’ demography. Student-t or Mann-Whitney-Wilcoxon tests were used to evaluate the differences between the two independent groups. The differences between independent categorical data groups were evaluated by Fisher exact test.

Age was categorized in 10 subgroups (Additional file 1: Table S1) for incidence analysis and 5 subgroups (<45, 45–54, 55–64, 65–74 and ≥75) for survival analysis. Crude and age standardized rates according to world population (CR and ASR (W), respectively) were calculated for incidence, prevalence and mortality. CR was represented as the number of patients per 100 000 inhabitants per year in Lithuania [16]. ASR (W) was defined as a weighted mean of the age-specific rates [17]; the weights were taken from the standard world population [18]. Incidence rate was defined as the number of new CML cases that occurred during a given time period [16]. Mortality rate was defined as a number of deaths from any cause during a given time period [16].

Relative survival rate (RSR) was defined as observed survival in CML group divided by the expected survival of a comparable group from general population [19]. Observed and expected survival were calculated using Kaplan-Meier [20] and Ederer II [21] method, respectively. One-, five- and ten-year RSRs with 95 % confidence intervals (95 % CI) were estimated. Survival time was calculated as the time from the date of diagnosis to death; 31st of December 2014 was set as censoring date for alive patients.

All statistics were performed by Statistical Analysis System (SAS) package version 9.2. A two-tailed *p*-value less than 0.05 was considered significant.

## Results

### Study population

From January 1st 2000 to December 31st 2013, 601 patients newly diagnosed with CML were included in the HESS registry. None of them were lost to follow-up. The median follow-up time for alive patients was 74 months (range 13–172). The genetic or cytogenetic confirmation of Ph + and/or BCR-ABL1 fusion status was available for 33 % of CML diagnoses made in 2000–2004, 83 % in 2005–2009 and 82 % in 2010–2013 period. Throughout the 14 year period male to female sex ratio was 1.09 with median age at diagnosis of 62 (range 14–93) and 63 (range 5–94) (*p* = 0.170), respectively. Older people were more likely to suffer from CML: 47 % of all CML cases were older than 65 years, although only 16 % of the Lithuanian population was older than 65 years (Table 1). The median age at diagnosis was 65 years at 2000–2004, 63 at 2005–2009 and 58 at 2010–2013 (*p* = 0.288). During the 2010–2013 period the share of CML diagnoses in people under 65 was higher (64 %) compared to two previous periods (49 % in 2000–2004, 53 % in 2005–2009) (*p* = 0.031).

**Table 1:** CML demographics in Lithuania in 2000–2013

Demographics	Calendar period						Total	
	2000–2004		2005–2009		2010–2013			
	N	%	N	%	N	%	N	%
Total	263	44	212	35	126	21	601	100
Age, years								
Median	65		63		58		62	
Range	5–94		9–90		17–94		5–94	
0–44	60	23	44	21	35	28	139	23
45–54	28	11	36	17	19	15	83	14
55–64	41	16	31	15	26	21	98	16
65–74	78	30	49	23	23	18	150	25
75+	56	21	52	25	23	18	131	22
Gender								
Male	139	53	108	51	66	52	313	52
Female	124	47	104	49	60	48	288	48
Hematopoietic stem cell transplantation								
Allogeneic	6	2	14^a^	7	7^b^	6	27	4

^a^2 patients with double haematopoietic stem cell transplant (HSCT)

^b^1 patient with double HSCT

### Incidence

The CR and ASR (W) of CML in Lithuanian population during the period of 2000–2013 was, accordingly, 1.28 (1.44 in males, 1.15 in females) and 0.88 (1.04 in males, 0.76 in females) (Table 2). Over the 14 year period of monitoring both metrics of CML incidence in Lithuania have steadily decreased. CR fell from 1.51 in 2000–2004 to 1.03 in 2010–2013. CML incidence increased with age. It was the lowest in the 0–14 age group (CR = 0.04) and the highest in ≥75 age group (CR = 4.36) (Additional file 1: Table S1). Although overall CML incidence was higher in men, greater male preponderance became evident only above the age of 60.

**Table 2:** CML epidemiology in Lithuania in 2000–2013

Epidemiology	Calendar period											
	2000–2004			2005–2009			2010–2013			2000–2013		
	Male	Female	All	Male	Female	All	Male	Female	All	Male	Female	All
Incidence rate												
CR	1.72	1.34	1.51	1.37	1.15	1.25	1.17	0.91	1.03	1.44	1.15	1.28
ASR (W)	1.20	0.87	1.00	1.00	0.72	0.86	0.88	0.65	0.75	1.04	0.76	0.88
Mortality rate												
CR	0.98	0.68	0.82	0.83	0.80	0.82	0.82	0.58	0.69	0.89	0.69	0.78
ASR (W)	0.62	0.38	0.47	0.61	0.46	0.52	0.52	0.29	0.38	0.59	0.38	0.46

*ASR (W)* age-standardized (weighted) rate, *CR* crude rate

### Accelerated phase, blast crisis and performance status

During the 2010–2013 period, accelerated phase (AP) and blast crisis (BC) data was available for 102 (81 %) and ECOG performance status for 92 (73 %) of newly diagnosed patients. At presentation 86 % were in CP, 12 % in AP and 2 % in BC. The median follow-up time for 2010–2013 cohort was 37 months (range 13–60 months). 1 patient progressed from CP to AP and 2 patients from AP to BC. None of the patients presenting with AP progressed into BC. At presentation, 92 % of patients below 75 years had ECOG performance status score of 0–1 (Additional file 1: Table S9). Only 22 % of ≥75 age group had ECOG 0–1.

### Mortality

Throughout the entire 2000–2013 period the average mortality CR and ASR (W) were 0.78 (range 0.26–1.08) and 0.46 (range 0.13–0.72), respectively. The average mortality CR and ASR (W) decreased from 0.82 to 0.69 and from 0.47 to 0.38, respectively, comparing 2000–2004 and 2010–2013 periods. Although there was no significant difference in crude male and female mortality rates (*p* = 0.095), men had a higher overall ASR (W) mortality (*p* = 0.014) (Table 2). Older age was a strong factor for higher mortality CR throughout all time periods (*p* < 0.001 for all periods) (Additional file 1: Table S4).

### Survival trends

The RSR improved with every calendar period of treatment. Overall, 1-, 5- and 10-year RSRs were 0.72, 0.49 and 0.36, respectively. RSR trends between males and females were similar throughout the entire follow-up period (*p* = 0.697) (Table 3). Age was an important predictor of RSR, with younger patients having significantly better RSR than elderly. 1-year RSR has markedly increased from 0.61 [95 % CI, 0.55–0.67] in 2000–2004 to 0.81 [95 % CI, 0.74–0.86] in 2005–2009, but plateaued at 0.81 [95 % CI, 0.74–0.86] during the 2010–2013 period (Table 3).

**Table 3:** One-, five- and ten-year RSR % of CML in Lithuania

Population	Calendar Period							
	2000–2004		2005–2009		2010–2013		2000–2013	
	RSR	95 % CI	RSR	95 % CI	RSR	95 % CI	RSR	95 % CI
1 year RSR								
All								
0–44 years	0.94	0.83–0.98	0.91	0.78–0.97	0.97	0.82–1.00	0.94	0.88–0.97
45–64 years	0.72	0.60–0.81	0.89	0.79–0.95	0.94	0.82–0.99	0.84	0.78–0.89
65–74 years	0.43	0.31–0.54	0.83	0.68–0.92	0.67	0.44–0.84	0.59	0.51–0.67
75+ years	0.35	0.22–0.49	0.57	0.41–0.71	0.49	0.26–0.69	0.46	0.37–0.55
Overall	0.61	0.55–0.67	0.81	0.74–0.86	0.81	0.74–0.86	0.72	0.69–0.76
Male								
0–44 years	0.91	0.73–0.91	0.85	0.64–0.94	0.96	0.72–1.00	0.90	0.81–0.95
45–64 years	0.70	0.52–0.82	0.90	0.72–0.97	1.00	-	0.84	0.74–0.90
65–74 years	0.39	0.24–0.53	0.81	0.59–0.93	0.81	0.46–0.96	0.58	0.46–0.68
75+ years	0.28	0.11–0.48	0.54	0.30–0.75	0.43	0.16–0.70	0.41	0.28–0.54
Overall	0.58	0.49–0.66	0.79	0.70–0.87	0.85	0.73–0.93	0.71	0.65–0.76
Female								
0–44 years	0.97	0.78–1.00	1.00	-	1.00	-	0.98	0.89–1.00
45–64 years	0.75	0.55–0.87	0.89	0.72–0.96	0.89	0.70–0.97	0.84	0.75–0.90
65–74 years	0.48	0.30–0.64	0.84	0.61–0.95	0.51	0.19–0.77	0.61	0.48–0.72
75+ years	0.41	0.23–0.59	0.59	0.38–0.76	0.56	0.20–0.84	0.51	0.38–0.62
Overall	0.65	0.55–0.73	0.82	0.73–0.89	0.80	0.67–0.89	0.74	0.68–0.79
5 year RSR								
All								
0–44 years	0.60	0.46–0.71	0.90	0.76–0.97	-	-	0.77	0.69–0.84
45–64 years	0.42	0.30–0.54	0.70	0.57–0.81	-	-	0.64	0.55–0.71
65–74 years	0.16	0.08–0.26	0.52	0.35–0.68	-	-	0.33	0.24–0.42
75+ years	0.14	0.05–0.29	0.09	0.02–0.22	-	-	0.14	0.07–0.23
Overall	0.33	0.27–0.40	0.55	0.47–0.63	N/R	N/R	0.49	0.45–0.54
Male								
0–44 years	0.53	0.34–0.70	0.83	0.61–0.94	-	-	0.74	0.62–0.83
45–64 years	0.44	0.27–0.61	0.74	0.53–0.89	-	-	0.66	0.53–0.77
65–74 years	0.14	0.05–0.28	0.50	0.27–0.73	-	-	0.33	0.21–0.46
75+ years	0.14	0.02–0.39	0.16	0.03–0.45	-	-	0.18	0.07–0.35
Overall	0.32	0.24–0.41	0.59	0.47–0.70	N/R	N/R	0.51	0.44–0.58
Female								
0–44 years	0.66	0.46–0.80	1.00	-	-	-	0.82	0.69–0.90
45–64 years	0.40	0.23–0.58	0.67	0.48–0.81	-	-	0.61	0.49–0.72
65–74 years	0.18	0.07–0.34	0.53	0.30–0.74	-	-	0.33	0.21–0.46
75+ years	0.14	0.04–0.34	0.05	0.00–0.21	-	-	0.11	0.04–0.23
Overall	0.35	0.26–0.44	0.52	0.41–0.63	N/R	N/R	0.48	0.41–0.55
10 year RSR								
All								
0–44 years	0.42	0.29–0.55	-	-	-	-	0.60	0.48–0.70
45–64 years	0.29	0.18–0.42	-	-	-	-	0.52	0.40–0.63
65–74 years	0.08	0.03–0.18	-	-	-	-	0.19	0.09–0.32
75+ years	-	-	-	-	-	-	-	-
Overall	0.21	0.16–0.27	N/R	N/R	N/R	N/R	0.36	0.31–0.42
Male								
0–44 years	0.38	0.21–0.56	-	-	-	-	0.58	0.41–0.72
45–64 years	0.33	0.17–0.52	-	-	-	-	0.53	0.37–0.69
65–74 years	0.04	0.00–0.18	-	-	-	-	0.11	0.01–0.36
75+ years	-	-	-	-	-	-	-	-
Overall	0.21	0.14–0.30	N/R	N/R	N/R	N/R	0.37	0.28–0.46
Female								
0–44 years	0.46	0.27–0.63	-	-	-	-	0.63	0.45–0.76
45–64 years	0.25	0.11–0.42	-	-	-	-	0.49	0.32–0.65
65–74 years	0.13	0.03–0.30	-	-	-	-	0.24	0.12–0.39
75+ years	-	-	-	-	-	-	-	-
Overall	0.21	0.14–0.30	N/R	N/R	N/R	N/R	0.36	0.28–0.44

**Fig. 1:**
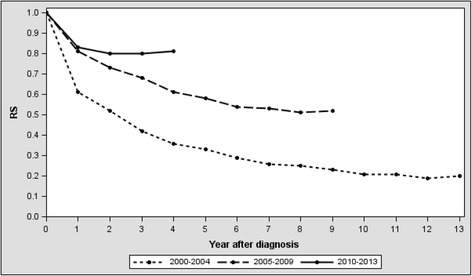
Relative survival rate by period of diagnosis

A 5-year CML RSR increased from 0.33 [95 % CI, 0.27–0.40] in 2000–2004 to 0.55 [95 % CI, 0.47–0.63] in 2005–2009 period in Lithuania. All patient groups, but those above 75, have demonstrated an increase in 5-year RSR. A 10-year RSR for the overall cohort was 0.36 [95 % CI, 0.31–0.42]. Figure 1 demonstrates how cumulative RSR changed with every calendar period. Interestingly, during the 2010–2013 period RSR started to increase beyond 2 years after the diagnosis.

Relative survival rate was calculated by diving the observed survival ratio by the expected survival ration of age-specific general population

### Treatment

**Fig. 2:**
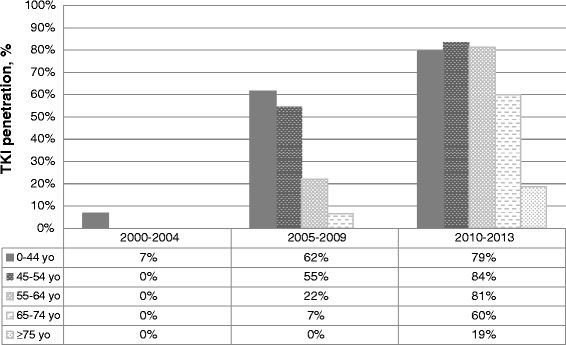
Tyrosine kinase inhibitor penetrance for treatment of CML in Lithuania

Figure 2 describes the TKI availability for CML patients in different calendar periods. TKI penetrance for CML patients grew from 1.5 % in 2000–2004 to 30.6 % in 2005–2009 and 69.1 % in 2010–2013. Imatinib was the only first line TKI for all study periods, which was guided by the national reimbursement policy. The increased availability of TKI treatment was largely limited to younger patient groups. In 2005–2009 49.5 % and in 2010–2013 81.0 % of patients younger than 65 were treated with TKIs. Until 2009 not a single patient ≥75 was treated with TKIs and even in the most recent period (2010–2013) the penetrance of TKIs in ≥75 patient group remained low (18.6 %). Across all three periods, 4 % of patients were treated with hematopoietic stem cell transplantation (4 %) (Table 1).

## Discussion

Imatinib generics are scheduled to enter US and European markets in early 2016 and the need for high quality population-level data to support better policy making is particularly increased [9, 22]. However, population-level reports reflecting the efficacy of CML treatment in TKI era are still scarce. Several available studies stem from haematology center-based registries or regional registries. Although valuable, they ignore the impact of out-of-region referral to treatment center and practice at local hospitals on presented data [14]. Entire country registry-based studies avoid these shortcomings, but cannot provide more detailed outcomes and have no control over the treatment and monitoring of the disease. Here we present one of few national registry CML epidemiology reports covering the population of the entire country. In addition, this is the first study demonstrating the CML treatment outcome inequalities between countries, owing to differences in the availability of the innovative treatment.

The age distribution of CML cases in Lithuania was virtually identical to the European averages, as summarized in the EUTOS population-based registry and HAEMACARE project reports [22, 23]. CML incidence CR in Lithuania was not stable throughout the observation period and decreased steadily from 1.51 in 2000–2004 period to 1.03 in 2010–2013. Cumulative CML incidence seen during the entire 2000–2013 period (CR 1.28) was slightly higher than CR of 1.10 seen in 48 European cancer registries in 2000–2002 and CR of 0.99 reported from EUTOS registry in 2008–2012 [24]. Meanwhile, reports from Swedish and UK registries both demonstrated even lower cumulative annual CML incidence (CR of 0.9) [12, 25]. Interestingly, considerably higher incidence metrics are seen in USA, where annual cumulative CML incidence CR has been consistently reported at 1.7–1.8 per 100000 population [26, 27].

Pathological and clinical diagnosis alone is known to sometimes misdiagnose Ph negative myeloproliferative neoplasias as CML [22]. BCR-ABL negative CML incidence was not available from the Lithuanian registry, but was previously reported to constitute 0.6 % of all CML cases in USA, 2 % in Italy and 4 % in France [3, 28, 29]. In Lithuanian HESS registry only a proportion of CML diagnoses were genetically confirmed, especially during the earliest study period. It is the drawback of our study, which probably explains the decreasing CML incidence over years, as share of genetically confirmed CML diagnoses increased. Therefore CR of 1.03 from the most recent 2010–2013 period is likely to represent the most accurate value of annual CML incidence in Lithuania. Likewise, a low percentage of genetically confirmed CML cases are seen in the SEER data and might underlie the observed CML incidence discrepancies between USA and Europe [22, 27]. In the latter only genetically confirmed CML diagnoses are included in the EUTOS registry. As much as 12 % of new CML diagnoses in Lithuania presented in the accelerated phase, whereas they comprised only 4 % of all new CML cases in Sweden and 5 % in Czech Republic and Slovakia [12, 14]. However, phase at presentation data was available only for the 2010–2013 period and larger numbers will be needed to draw a definitive conclusion of higher incidence of AP-CML in Lithuania compared to other countries.

Introduction of imatinib mesylate as a first line therapy has led to great improvements in prognosis for CML patients. Treatment results in IRIS trial demonstrated a 89 % overall 5-year survival for patients aged 18–70 and treated with imatinib as a first-line agent [30]. Similarly high long-term survival rates were also demonstrated in centers of excellence for CML care [31, 32]. Since oral administration of imatinib significantly reduced the complexity of care for CML patients, the treatment may be decentralized and provided outside teaching hospitals. However, a study by Lauseker et al*.* found that in Germany outcomes for patients treated outside teaching hospitals are markedly inferior to those treated in academic centers [33]. In Sweden academic center catchment areas also had a tendency to give superior CML treatment results to other regions, though not at a statistically significant level [12]. Thus national survival averages are likely to be worse than survival reports from sole specialty centers, or regional registries, based around tertiary haematology center.

In Lithuania, country with 3 million inhabitants, CML is treated in 5 hospitals, 2 of which are teaching hospitals. Prior to TKI entry, CML patients in Lithuania were treated with hydroxyurea and IFNα, while only occasional eligible patient received a haematological stem cell transplant (HSCT). 5-year RSR increased from 33 % in 2000–2004 to 55 % in 2005–2009, when TKIs became partially available in Lithuania. Recently EUROCARE-5 project reported haematological cancer RSRs for different European regions [34]. Here Lithuania along with Estonia, Slovakia, Poland and Bulgaria constituted the Eastern Europe region. Throughout 2000–2004 and 2005–2009 periods, 5-year CML RSR in Lithuania remained above the Eastern European registry averages, at overall European average, but below the RSR averages seen in Sweden (Table 4). Every age group 5-year RSR averages also remained lower than those reported from UK, USA and Girona province of Spain [13, 35, 36]. Unfortunately, UK data in Pulte et al*.* study may be compounded by CML registration inaccuracies [37]. Smith et al*.* report demonstrates that the actual 5-year RSR in UK is probably significantly higher (0.89 for 2004–2011 period) and no worse than CML RSR seen in Sweden [36]. Similarly to other studies, the biggest 5-year RSR improvements in Lithuania were evident in patient groups <75 [2, 3]. 1-year (RSR 0.46, 95 % CI 0.37–0.55) and 5-year (RSR 0.14, 95 % CI 0.07–0.23) RSR in patients ≥75 remained low throughout the entire 2000–2013 period.

**Table 4:** 5-year RSR for CML patients from registry data stratified by region of origin and time period

Source	Time period	5 Year RSR % (95 % CI)
Europe	2000–2002	33.8 (32.2–35.4)
Sant et al. 2014 [34]	2003–2005	45.7 (43.9–47.5)
	2006–2008	54.4 (52.5–56.2)
Eastern Europe^a^	2000–2002	26.4 (22.4–30.6)
Sant et al. 2014 [34]	2003–2005	28.6 (25.0–32.4)
	2006–2008	35.3 (30.9–39.7)
Sweden	2001–2008	80 (75–83)
Bjorkholm et al. 2011 [2]		
Lithuania	2000–2004	33 (27–40)
Our data	2005–2009	55 (47–60)

^a^Eastern Europe region: Lithuania, Estonia, Poland, Slovakia and Bulgaria

Five-year RSR for patients newly diagnosed with CML in 2010–2013 is not available yet, but cumulative 4-year RS for this period was >0.80. Relative survival for this latest Lithuanian patient cohort is approaching the 5-year RSR recorded in French cancer registries (RSR 0.83 in 2000–2009) and Swedish national registry (RSR 0.80 in 2001–2008) in the early years of TKI utilization [2, 3]. Interestingly, the patient population diagnosed with CML during the 2010–2013 period, started showing an upward RSR trend two years after the diagnosis. This finding is in line with results published by Gambacorti-Passerini et al*.* who show that people treated with imatinib and in cytogenic remission for >2 years, carry only 4.8 % annual overall mortality, which is similar to matched general population [38]. It is possible that imatinib has a long term cardiovascular protective effect [39], though opposite claims have also been published [40].

The emergence of effective treatment has also sparkled enthusiasm in standardizing the CML referral pathways, formulating explicit treatment guidelines and employing the newest molecular disease monitoring and prognostication techniques, which have potentially led to the improvement of CML patient care and survival. Yet TKI penetrance is probably the sole most important determinant of CML survival on a country level. In Lithuania the penetrance of TKI treatment was largely determined by national reimbursement policy. Owing to healthcare resource restrictions, here patented cancer therapies have longer availability latency than in Western Europe. While in multiple Western economies imatinib entered national CML treatment guidelines as a first line CML treatment in 2001-2, imatinib became partially available in Lithuania only in 2005. During the 2005–2009 period TKI treatment was reserved only for the youngest patients: 58 % patients aged <55 and only 8 % patients ≥55 received TKIs. Only from 2011 all newly diagnosed CML patients were funded to have imatinib as a first line treatment, but even during the 2010–2013 period of CML diagnosis, in Lithuania only 69 % of patients were treated with TKIs.

A noteworthy study was recently published from another less affluent country - Bosnia and Herzegovina – demonstrating that many patients experience a delay in receiving the TKI treatment and therefore have worse cytogenetic and molecular remission rates [41]. Individual case TKI reimbursement system seen Bosnia functioned in Lithuania between 2005 and 2011 and here the argument that delay in administering TKIs could lead to inferior outcomes is also valid.

Although excellent CML survival rates have been reported from regional population databases in France and UK, so far no other country has reported better entire country population CML survival results than Sweden [3, 12, 36]. Hoglund et al*.* data contradicts the criticism that clinical trial treatment efficacy results cannot be expected in general population [11]. With nearly complete CML population coverage with TKIs, survival for chronic phase CML patients aged ≤60 in Sweden was close to survival of general population [12]. Since 2004 > 85 % CML patients have been receiving TKIs as a first-line treatment in Sweden [12]. A French report showed that 93 % of patients diagnosed with CML during 2000–2009 period were treated with TKIs [3]. Interestingly, in some countries TKI availability is not a correlate of the economic output. Due to loose regulations and easily available cheap imatinib generics, some Pacific-Asian countries have even higher TKI coverage than some Western countries that pay the full patented drug price [42].

Even during 2010–2013 period the accessibility of TKIs for patients ≥75 was much smaller (18 %) than for younger population (81 % of <65 years). Likewise, in USA significantly fewer elderly patients, when compared to younger patients, were given TKIs during the 2003–2005 observation period, though these rates would have certainly increased by now [43]. Meanwhile, Swedish national registry report shows that during 2001–2008 period >80 % of CML patients aged ≥75 received TKIs [12]. Overall CML survival, when treated with TKIs, was shown to be more dependent on the number of comorbidities than on patient’s age [44]. Our data demonstrates that patients ≥75 presenting with CML have markedly worse ECOG performance status than younger patients and this may underlie physicians’ decision to withhold the TKI treatment. However, only 60 % of 65–74 year old CML patients, who had much better performance status, were prescribed imatinib. There may well be a bias among doctors to withhold the expensive treatment from elderly patients with a view of reserving it for the younger. However, studies show that elderly CML patients benefit from TKI treatment nearly as much as younger patients and age has no objective role as a selection criteria for the TKI treatment [45].

## Conclusion

In Lithuania crude CML incidence matched the European averages once strict genetic diagnostics criteria were implemented. Relative CML survival improved from 2000–2004 to 2010–2013 period and was paralleled by the increasing availability of TKI treatment. CML patients in Lithuania had better relative survival than the Eastern European average, but lower than CML patients in more affluent countries, where TKI penetrance was higher. Patients above 75 years rarely received TKIs and their relative survival remained low throughout the observation period.

## Additional file

Additional file 1: Supplemetary material providing detailed data on age group-specific CR and ASR (W) of CML incidence and mortality, time-period specific Kaplan-Meier survival curves and ECOG performance status of different patient groups. (PDF 766 kb)

## Abbreviations

ASR(W): age-standardized rate (weighted)
CML: chronic myelogenous leukaemia
CR: crude rate
HESS: Haematological disease monitoring system
HSCT: Haematological stem cell transplant
RSR: relative survival rate
TKI: tyrosine kinase inhibitor
