# Supplementary material for: Chronic myeloid leukemia incidence, survival and accessibility of tyrosine kinase inhibitors: a report from population-based Lithuanian haematological disease registry 2000–2013
Source: BMC Cancer. 2016 Mar 8;16:198. doi: 10.1186/s12885-016-2238-9 (PMC4782571; doi:10.1186/s12885-016-2238-9)
Supplement: Additional file 1: — Supplemetary material providing detailed data on age group-specific CR and ASR (W) of CML incidence and mortality, time-period specific Kaplan-Meier survival curves and ECOG performance status of different patient groups. (PDF 766 kb) [file 12885_2016_2238_MOESM1_ESM.pdf]

Chronic myeloid leukemia incidence, survival and accessibility of tyrosine kinase inhibitors: a report from population-based Lithuanian haematological disease registry 2000-2013

Tumas Beinortas, Ilma Tavorienė, Tadas Žvirblis, Rolandas Gerbutavičius, Mindaugas Jurgutis, Laimonas Griškevičius

Supplement

Lithuanian HESS registry

Lithuania has a national haematological disease monitoring system (HESS), which collects data from 2000 and covers the entire country, with a population of 3 million. Patients with haematological malignancies are managed in 5 centres across the country and all physicians and pathologists are obliged to report all newly diagnosed CML cases to HESS Registry. HESS contains data on age, sex, ICD-10 code, date of diagnosis, clinical symptoms, laboratory test, risk group, treatment, Ph and BCR-ABL status (both mandatory from 2010) of CML. Through unique personal ID, HESS is also linked to the national death registry, which allows further validation of data. All Lithuanians are covered by national healthcare insurance and haematological diseases are treated in public healthcare system. Therefore underreporting to HESS registry is unlikely. All patients, who were diagnosed with CML (ICD-10 code 92.1) between January 1st 2000 and December 31st 2013, were entered into the study. There was no age restriction or other exclusion criteria.

Statistical methods

Crude and age standardize rates according to world population (CR and ASR (W), respectively) were calculated for incidence, prevalence and mortality. CR was represented as the number of patients per 100 000 inhabitants per year in Lithuania [1]. ASR (W) was defined as a weighted mean of the age-specific rates [2]; the weights were taken from the standard world population [3]. Incidence rate was defined as the number of new CML cases that occurred during a given time period [1]. Mortality rate was defined as a number of deaths from any cause during a given time period [1].

Relative survival rate (RSR) was defined as observed survival in CML group divided by the expected survival of a comparable group from general population [4]. One-, five- and ten-year RSRs with 95% confidence intervals (95% CI) were estimated. Survival time was calculated as the time from the date of diagnosis to death; 31st of December 2014 was set as censoring date for alive patients.

Survival trends were evaluated by Kaplan-Meier method. Log-rank test was used evaluate statistical difference between Kaplan-Meier curves.

All statistics were performed by Statistical Analysis System (SAS) package version 9.2. A two-tailed p-value less than 0.05 was considered significant.

Table S1. Incidence Crude Rate of Chronic Myeloid Leukemia according to age group (per 100 000 per year) (10 age groups).

Age groups	Age group, years										Overall
	0-14	15-39	40-44	45-49	50-54	55-59	60-64	65-69	70-74	75+	
**All population**											
**2000**	0.00	0.31	0.74	1.37	2.03	2.18	2.18	4.18	4.83	5.89	**1.31**
**2001**	0.00	0.95	1.87	0.46	2.54	2.74	3.28	3.60	8.32	4.31	**1.72**
**2002**	0.00	0.48	0.73	0.45	1.48	1.72	2.18	4.81	4.81	4.65	**1.24**
**2003**	0.16	0.88	2.19	1.74	0.49	4.09	2.75	6.65	6.20	8.37	**2.08**
**2004**	0.00	0.40	2.20	0.41	2.43	1.14	0.56	2.41	4.86	5.30	**1.22**
**Average (2000-2004)**	**0.03**	**0.60**	**1.55**	**0.89**	**1.79**	**2.37**	**2.19**	**4.33**	**5.80**	**5.70**	**1.51**
**2005**	0.00	0.40	0.74	0.80	0.97	2.78	0.58	2.42	3.48	5.71	**1.11**
**2006**	0.18	0.73	1.15	2.32	1.92	1.63	0.59	4.87	4.18	2.77	**1.38**
**2007**	0.19	0.32	0.39	1.89	2.35	1.58	3.72	3.66	4.88	6.28	**1.54**
**2008**	0.00	0.98	0.40	1.13	1.82	1.56	3.18	1.84	0.70	5.24	**1.31**
**2009**	0.00	0.41	0.00	1.14	0.87	1.04	1.25	3.15	2.80	3.42	**0.93**
**Average (2005-2009)**	**0.07**	**0.57**	**0.54**	**1.46**	**1.59**	**1.72**	**1.86**	**3.19**	**3.21**	**4.68**	**1.25**
**2010**	0.00	0.43	0.84	2.34	0.00	2.08	1.83	2.59	1.40	2.52	**0.97**
**2011**	0.00	0.90	1.38	0.88	1.28	1.62	2.42	0.68	1.39	3.15	**1.16**
**2012**	0.00	0.72	1.40	0.91	1.68	2.65	1.77	2.15	6.28	1.93	**1.37**
**2013**	0.00	0.42	0.95	0.47	0.42	1.53	0.58	0.73	0.71	1.51	**0.61**
**Average (2010-2013)**	**0.00**	**0.62**	**1.14**	**1.15**	**0.85**	**1.97**	**1.65**	**1.54**	**2.45**	**2.28**	**1.03**
**Average (2000-2013)**	**0.04**	**0.59**	**1.07**	**1.16**	**1.45**	**2.02**	**1.92**	**3.12**	**3.92**	**4.36**	**1.28**
**Males**											
**2000**	0.00	0.16	0.77	0.96	4.42	3.72	3.89	9.07	5.72	7.49	**1.59**
**2001**	0.00	0.47	2.33	0.97	3.33	2.49	5.21	3.04	17.26	7.54	**1.90**
**2002**	0.00	0.47	0.00	0.95	1.08	1.30	1.30	9.15	3.77	5.42	**1.11**
**2003**	0.00	1.43	3.77	2.74	0.00	3.98	2.62	10.75	5.67	13.69	**2.48**
**2004**	0.00	0.48	2.27	0.00	3.17	2.60	0.00	4.61	9.53	8.35	**1.50**
**Average (2000-2004)**	**0.00**	**0.60**	**1.83**	**1.12**	**2.40**	**2.82**	**2.60**	**7.32**	**8.39**	**8.50**	**1.72**
**2005**	0.00	0.48	0.76	0.84	1.05	2.53	1.38	3.08	3.84	8.21	**1.13**
**2006**	0.00	0.48	1.57	2.46	2.07	1.23	0.00	3.11	5.79	4.75	**1.20**
**2007**	0.37	0.64	0.00	2.40	3.06	2.39	4.47	7.81	11.61	9.22	**2.10**
**2008**	0.00	1.13	0.82	0.79	1.98	1.18	3.05	3.15	0.00	5.98	**1.28**
**2009**	0.00	0.65	0.00	2.39	0.00	1.17	1.51	4.87	1.95	7.33	**1.16**
**Average (2005-2009)**	**0.07**	**0.68**	**0.63**	**1.78**	**1.63**	**1.70**	**2.08**	**4.40**	**4.64**	**7.10**	**1.37**
**2010**	0.00	0.51	0.86	0.82	0.00	2.34	1.47	3.33	3.92	7.20	**1.11**
**2011**	0.00	1.39	1.91	0.91	0.92	1.20	2.87	1.75	0.00	4.04	**1.29**
**2012**	0.00	0.41	1.94	0.95	1.80	2.36	2.79	1.83	9.60	2.63	**1.38**
**2013**	0.00	0.83	0.99	0.00	0.00	1.13	1.38	1.86	1.95	3.87	**0.88**
**Average (2010-2013)**	**0.00**	**0.79**	**1.43**	**0.67**	**0.68**	**1.76**	**2.13**	**2.19**	**3.87**	**4.44**	**1.17**

**Average (2000-2013)**	**0.03**	**0.68**	**1.29**	**1.23**	**1.64**	**2.12**	**2.28**	**4.82**	**5.76**	**6.84**	**1.44**
**Females**											
**2000**	0.00	0.47	0.72	1.74	0.00	0.97	0.94	0.99	4.32	5.26	**1.07**
**2001**	0.00	1.43	1.44	0.00	1.88	2.93	1.89	3.96	3.26	3.02	**1.56**
**2002**	0.00	0.48	1.41	0.00	1.83	2.04	2.82	1.98	5.41	4.34	**1.35**
**2003**	0.33	0.32	0.70	0.83	0.90	4.18	2.84	3.98	6.50	6.22	**1.74**
**2004**	0.00	0.32	2.13	0.79	1.80	0.00	0.97	0.99	2.18	4.06	**0.98**
**Average (2000-2004)**	**0.07**	**0.60**	**1.28**	**0.67**	**1.28**	**2.02**	**1.89**	**2.38**	**4.33**	**4.58**	**1.34**
**2005**	0.00	0.32	0.72	0.76	0.90	2.98	0.00	1.99	3.28	4.68	1.10
**2006**	0.37	0.98	0.75	2.20	1.78	1.94	1.02	6.00	3.27	1.95	1.55
**2007**	0.00	0.00	0.77	1.44	1.75	0.94	3.19	1.00	1.09	5.06	1.05
**2008**	0.00	0.82	0.00	1.43	1.69	1.86	3.28	1.01	1.09	4.93	1.34
**2009**	0.00	0.17	0.00	0.00	1.62	0.93	1.07	2.06	3.27	1.81	0.73
**Average (2005-2009)**	**0.07**	**0.46**	**0.45**	**1.17**	**1.55**	**1.73**	**1.71**	**2.41**	**2.40**	**3.69**	**1.15**
**2010**	0.00	0.35	0.81	3.75	0.00	1.87	2.08	2.12	0.00	0.59	0.85
**2011**	0.00	0.40	0.89	0.84	1.60	1.95	2.09	0.00	2.18	2.79	1.04
**2012**	0.00	1.04	0.90	0.87	1.57	2.88	1.02	2.35	4.38	1.63	1.37
**2013**	0.00	0.00	0.92	0.89	0.79	1.85	0.00	0.00	0.00	0.54	0.38
**Average (2010-2013)**	**0.00**	**0.45**	**0.88**	**1.59**	**0.99**	**2.14**	**1.30**	**1.12**	**1.64**	**1.39**	**0.91**
**Average (2000-2013)**	**0.05**	**0.51**	**0.87**	**1.11**	**1.29**	**1.95**	**1.66**	**2.03**	**2.87**	**3.35**	**1.15**

Table S2. Incidence Crude Rate of Chronic Myeloid Leukemia according to age group (per 100 000 per year) (5 age groups).

Age groups	Age group, years					Overall
	0-17	18-45	46-60	61-75	76+	
**All population**						
**2000**	0.12	0.42	1.71	3.77	6.85	**1.31**
**2001**	0.00	1.21	2.04	5.05	4.13	**1.72**
**2002**	0.00	0.57	1.19	4.00	5.08	**1.24**
**2003**	0.13	1.28	2.02	5.46	8.09	**2.08**
**2004**	0.00	0.78	1.32	2.74	5.27	**1.22**
**Average (2000-2004)**	**0.05**	**0.85**	**1.66**	**4.20**	**5.88**	**1.51**
**2005**	0.14	0.43	1.45	2.13	6.23	**1.11**
**2006**	0.14	0.93	2.05	3.25	2.51	**1.38**
**2007**	0.15	0.58	1.85	3.95	6.34	**1.54**
**2008**	0.15	1.02	1.21	2.00	5.69	**1.31**
**2009**	0.16	0.29	1.04	2.70	3.24	**0.93**
**Average (2005-2009)**	**0.15**	**0.65**	**1.52**	**2.81**	**4.80**	**1.25**
**2010**	0.00	0.52	1.66	2.04	2.23	**0.97**
**2011**	0.18	0.96	1.41	1.81	2.55	**1.16**
**2012**	0.18	0.89	1.72	3.18	2.08	**1.37**
**2013**	0.19	0.45	0.78	0.68	1.68	**0.61**
**Average (2010-2013)**	**0.14**	**0.71**	**1.39**	**1.93**	**2.14**	**1.03**
**Average (2000-2013)**	**0.11**	**0.74**	**1.53**	**3.06**	**4.43**	**1.28**
**Male**						
**2000**	0.00	0.29	3.01	6.47	8.57	**1.59**
**2001**	0.00	0.86	2.61	8.05	6.38	**1.90**
**2002**	0.00	0.43	1.11	4.81	6.14	**1.11**
**2003**	0.00	2.15	1.84	7.48	11.69	**2.48**
**2004**	0.00	0.86	1.80	4.31	9.46	**1.50**
**Average (2000-2004)**	**0.00**	**0.92**	**2.07**	**6.22**	**8.45**	**1.72**
**2005**	0.27	0.43	1.41	2.73	9.30	**1.13**
**2006**	0.00	0.72	2.06	3.34	3.57	**1.20**
**2007**	0.29	0.87	2.02	7.94	10.38	**2.10**
**2008**	0.29	1.17	0.99	2.30	6.72	**1.28**
**2009**	0.30	0.44	1.29	2.90	8.20	**1.16**
**Average (2005-2009)**	**0.23**	**0.73**	**1.55**	**3.84**	**7.63**	**1.37**
**2010**	0.00	0.60	0.95	3.48	6.30	**1.11**
**2011**	0.34	1.40	1.01	1.74	4.49	**1.29**
**2012**	0.00	0.72	2.02	4.07	2.92	**1.38**
**2013**	0.36	0.73	0.34	1.75	4.38	**0.88**
**Average (2010-2013)**	**0.18**	**0.86**	**1.08**	**2.76**	**4.52**	**1.17**
**Average (2000-2013)**	**0.13**	**0.83**	**1.61**	**4.38**	**7.04**	**1.44**
**Female**						

**2000**	0.24	0.56	0.63	2.06	6.15	**1.07**
**2001**	0.00	1.54	1.56	3.12	3.27	**1.56**
**2002**	0.00	0.70	1.25	3.47	4.68	**1.35**
**2003**	0.26	0.42	2.18	4.15	6.71	**1.74**
**2004**	0.00	0.70	0.92	1.73	3.65	**0.98**
**Average (2000-2004)**	**0.10**	**0.78**	**1.31**	**2.91**	**4.89**	**1.34**
**2005**	0.00	0.43	1.49	1.75	5.04	**1.10**
**2006**	0.29	1.14	2.05	3.19	2.10	**1.55**
**2007**	0.00	0.29	1.71	1.43	4.76	**1.05**
**2008**	0.00	0.87	1.40	1.82	5.29	**1.34**
**2009**	0.00	0.15	0.83	2.56	1.29	**0.73**
**Average (2005-2009)**	**0.06**	**0.58**	**1.50**	**2.15**	**3.70**	**1.15**
**2010**	0.00	0.45	2.29	1.11	0.62	**0.85**
**2011**	0.00	0.52	1.75	1.86	1.78	**1.04**
**2012**	0.37	1.07	1.46	2.61	1.74	**1.37**
**2013**	0.00	0.18	1.16	0.00	0.59	**0.38**
**Average (2010-2013)**	**0.09**	**0.56**	**1.67**	**1.40**	**1.18**	**0.91**
**Average (2000-2013)**	**0.08**	**0.64**	**1.48**	**2.20**	**3.41**	**1.15**

Table S3. Incidence ASR (W) of Chronic Myeloid Leukemia (per 100 000 per year).

Overall	All population	Males	Females
**2000**	0.82	1.05	0.67
**2001**	1.19	1.30	1.18
**2002**	0.79	0.77	0.83
**2003**	1.40	1.83	1.05
**2004**	0.80	1.05	0.63
**Average (2000-2004)**	**1.00**	**1.20**	**0.87**
**2005**	0.72	0.80	0.66
**2006**	1.02	0.87	1.17
**2007**	0.99	1.50	0.59
**2008**	0.96	1.02	0.91
**2009**	0.60	0.83	0.42
**Average (2005-2009)**	**0.86**	**1.00**	**0.75**
**2010**	0.69	0.79	0.65
**2011**	0.87	1.09	0.67
**2012**	0.96	0.95	1.01
**2013**	0.46	0.69	0.27
**Average (2010-2013)**	**0.75**	**0.88**	**0.65**
**Overall**	**0.88**	**1.04**	**0.76**

Table S4. Mortality Crude Rate of Chronic Myeloid Leukemia according to age group (per 100 000 per year).

Age groups	Age group, years										Overall
	0-14	15-39	40-44	45-49	50-54	55-59	60-64	65-69	70-74	75+	
**All population**											
**2000**	0.00	0.00	0.00	0.00	0.51	0.00	0.54	1.19	1.38	1.61	**0.26**
**2001**	0.00	0.24	0.00	0.00	0.00	1.10	2.74	3.60	8.32	3.23	**0.98**
**2002**	0.00	0.08	0.37	0.00	0.99	0.57	0.00	4.21	2.75	4.65	**0.72**
**2003**	0.00	0.32	0.00	0.87	0.98	0.58	2.20	3.02	4.13	6.40	**1.07**
**2004**	0.00	0.24	0.37	0.00	0.97	3.43	1.13	1.21	3.47	7.71	**1.08**
**Average (2000-2004)**	**0.00**	**0.18**	**0.15**	**0.17**	**0.69**	**1.14**	**1.32**	**2.65**	**4.01**	**4.72**	**0.82**
**2005**	0.17	0.08	0.74	0.40	0.00	1.11	2.31	4.83	2.79	3.33	**0.88**
**2006**	0.00	0.24	0.76	0.39	0.48	0.54	1.19	0.00	3.48	2.77	**0.62**
**2007**	0.19	0.40	1.57	0.38	0.94	2.11	1.24	1.83	3.49	3.14	**1.01**
**2008**	0.00	0.33	0.00	1.50	0.91	1.04	0.64	1.84	3.50	5.24	**0.98**
**2009**	0.00	0.00	0.41	0.00	0.44	1.04	1.88	1.89	1.40	3.85	**0.63**
**Average (2005-2009)**	**0.07**	**0.21**	**0.70**	**0.53**	**0.55**	**1.17**	**1.45**	**2.08**	**2.93**	**3.67**	**0.82**
**2010**	0.00	0.09	0.42	0.78	1.69	0.52	0.00	3.24	2.10	5.46	**0.91**
**2011**	0.00	0.10	0.92	0.44	0.00	0.54	1.21	2.05	4.16	2.76	**0.76**
**2012**	0.00	0.10	0.00	0.00	0.00	0.53	1.18	1.43	2.79	3.47	**0.64**
**2013**	0.00	0.10	1.43	0.00	0.00	0.00	0.58	1.46	0.71	1.89	**0.44**
**Average (2010-2013)**	**0.00**	**0.10**	**0.69**	**0.31**	**0.42**	**0.40**	**0.74**	**2.05**	**2.44**	**3.40**	**0.69**
**Average (2000-2013)**	**0.03**	**0.17**	**0.50**	**0.34**	**0.56**	**0.94**	**1.20**	**2.27**	**3.18**	**3.96**	**0.78**
**Males**											
**2000**	0.00	0.00	0.00	0.00	1.10	0.00	0.00	3.02	1.91	3.75	**0.37**
**2001**	0.00	0.16	0.00	0.00	0.00	1.25	5.21	3.04	17.26	5.65	**1.23**
**2002**	0.00	0.16	0.76	0.00	2.16	0.00	0.00	9.15	1.89	7.23	**0.93**
**2003**	0.00	0.16	0.00	0.00	1.06	0.00	5.23	7.68	5.67	10.26	**1.24**
**2004**	0.00	0.32	0.00	0.00	1.06	5.20	1.34	3.07	3.81	10.02	**1.12**
**Average (2000-2004)**	**0.00**	**0.16**	**0.15**	**0.00**	**1.08**	**1.29**	**2.36**	**5.19**	**6.11**	**7.38**	**0.98**
**2005**	0.00	0.16	1.53	0.00	0.00	1.27	0.00	7.71	3.84	4.93	**0.88**
**2006**	0.00	0.32	0.78	0.82	1.04	1.23	1.42	0.00	1.93	4.75	**0.70**
**2007**	0.37	0.80	1.61	0.00	1.02	2.39	1.49	3.12	5.81	3.07	**1.21**
**2008**	0.00	0.32	0.00	2.37	1.98	1.18	1.53	0.00	5.85	4.49	**0.96**
**2009**	0.00	0.00	0.00	0.00	0.00	0.00	0.00	3.24	1.95	7.33	**0.51**
**Average (2005-2009)**	**0.07**	**0.32**	**0.78**	**0.64**	**0.81**	**1.21**	**0.89**	**2.81**	**3.88**	**4.91**	**0.85**
**2010**	0.00	0.17	0.86	1.63	0.92	1.17	0.00	5.00	3.92	12.97	**1.31**
**2011**	0.00	0.20	0.00	0.00	0.00	1.20	0.00	3.50	5.72	2.69	**0.64**
**2012**	0.00	0.20	0.00	0.00	0.00	0.00	2.79	1.83	1.92	5.26	**0.65**
**2013**	0.00	0.21	1.98	0.00	0.00	0.00	1.38	3.73	0.00	3.87	**0.66**
**Average (2010-2013)**	**0.00**	**0.20**	**0.71**	**0.41**	**0.23**	**0.59**	**1.04**	**3.52**	**2.89**	**6.20**	**0.82**
**Average (2000-2013)**	**0.03**	**0.23**	**0.54**	**0.34**	**0.74**	**1.06**	**1.46**	**3.86**	**4.39**	**6.16**	**0.89**
**Females**											
**2000**	0.00	0.00	0.00	0.00	0.00	0.00	0.94	0.00	1.08	0.75	**0.16**
**2001**	0.00	0.32	0.00	0.00	0.00	0.98	0.94	3.96	3.26	2.26	**0.76**
**2002**	0.00	0.00	0.00	0.00	0.00	1.02	0.00	0.99	3.24	3.61	**0.54**
**2003**	0.00	0.48	0.00	1.65	0.90	1.05	0.00	0.00	3.25	4.84	**0.92**
**2004**	0.00	0.16	0.71	0.00	0.90	2.05	0.97	0.00	3.27	6.77	**1.04**

Age groups	Age group, years										Overall
	0-14	15-39	40-44	45-49	50-54	55-59	60-64	65-69	70-74	75+	
**Average (2000-2004)**	**0.00**	**0.19**	**0.14**	**0.33**	**0.36**	**1.02**	**0.57**	**0.99**	**2.82**	**3.65**	**0.68**
**2005**	0.36	0.00	0.00	0.76	0.00	0.99	3.97	2.98	2.19	2.68	**0.88**
**2006**	0.00	0.16	0.75	0.00	0.00	0.00	1.02	0.00	4.37	1.95	**0.55**
**2007**	0.00	0.00	1.53	0.72	0.87	1.88	1.06	1.00	2.18	3.17	**0.83**
**2008**	0.00	0.33	0.00	0.72	0.00	0.93	0.00	3.02	2.19	5.55	**1.00**
**2009**	0.00	0.00	0.80	0.00	0.81	1.86	3.22	1.03	1.09	2.41	**0.73**
**Average (2005-2009)**	**0.07**	**0.10**	**0.62**	**0.44**	**0.34**	**1.13**	**1.85**	**1.61**	**2.40**	**3.15**	**0.80**
**2010**	0.00	0.00	0.00	0.00	2.35	0.00	0.00	2.12	1.09	2.37	**0.57**
**2011**	0.00	0.00	1.77	0.84	0.00	0.00	2.09	1.12	3.26	2.79	**0.86**
**2012**	0.00	0.00	0.00	0.00	0.00	0.96	0.00	1.17	3.29	2.72	**0.62**
**2013**	0.00	0.00	0.92	0.00	0.00	0.00	0.00	0.00	1.11	1.07	**0.25**
**Average (2010-2013)**	**0.00**	**0.00**	**0.67**	**0.21**	**0.59**	**0.24**	**0.52**	**1.10**	**2.19**	**2.24**	**0.58**
**Average (2000-2013)**	**0.03**	**0.10**	**0.46**	**0.33**	**0.42**	**0.84**	**1.02**	**1.24**	**2.49**	**3.07**	**0.69**

Table S5. Mortality Crude Rate of Chronic Myeloid Leukemia according to age group (per 100 000 per year) (5 age groups).

Age groups	Age group, years					Overall
	0-17	18-45	46-60	61-75	76+	
**All population**						
**2000**	0.00	0.00	0.17	1.05	1.87	**0.26**
**2001**	0.12	0.14	0.51	4.84	2.95	**0.98**
**2002**	0.00	0.14	0.51	2.31	5.08	**0.72**
**2003**	0.00	0.35	0.67	3.36	6.47	**1.07**
**2004**	0.00	0.28	1.32	2.11	7.90	**1.08**
**Average (2000-2004)**	**0.02**	**0.18**	**0.64**	**2.73**	**4.85**	**0.82**
**2005**	0.14	0.21	0.65	3.20	3.63	**0.88**
**2006**	0.00	0.36	0.47	1.73	2.51	**0.62**
**2007**	0.15	0.72	0.93	2.19	3.42	**1.01**
**2008**	0.15	0.29	1.06	2.00	5.69	**0.98**
**2009**	0.00	0.07	0.74	1.57	3.71	**0.63**
**Average (2005-2009)**	**0.09**	**0.33**	**0.77**	**2.14**	**3.79**	**0.82**
**2010**	0.16	0.15	0.90	1.81	5.81	**0.91**
**2011**	0.00	0.26	0.62	2.49	2.13	**0.76**
**2012**	0.00	0.09	0.16	1.82	3.74	**0.64**
**2013**	0.00	0.36	0.00	0.91	2.10	**0.44**
**Average (2010-2013)**	**0.04**	**0.22**	**0.42**	**1.76**	**3.45**	**0.69**
**Average (2000-2013)**	**0.05**	**0.25**	**0.62**	**2.24**	**4.07**	**0.78**
**Male**						
**2000**	0.00	0.00	0.38	1.62	4.29	**0.37**
**2001**	0.00	0.14	0.75	8.05	4.25	**1.23**
**2002**	0.00	0.29	0.74	3.74	8.19	**0.93**
**2003**	0.00	0.14	0.37	6.95	9.74	**1.24**
**2004**	0.00	0.29	1.80	2.70	11.35	**1.12**
**Average (2000-2004)**	**0.00**	**0.17**	**0.81**	**4.61**	**7.56**	**0.98**
**2005**	0.00	0.43	0.35	3.83	5.58	**0.88**
**2006**	0.00	0.43	1.03	1.67	3.57	**0.70**
**2007**	0.29	1.01	1.01	3.40	3.46	**1.21**
**2008**	0.29	0.29	1.64	2.30	5.04	**0.96**
**2009**	0.00	0.00	0.00	1.74	8.20	**0.51**
**Average (2005-2009)**	**0.12**	**0.43**	**0.81**	**2.59**	**5.17**	**0.85**
**2010**	0.32	0.30	0.95	2.90	14.18	**1.31**
**2011**	0.00	0.18	0.34	3.48	1.50	**0.64**
**2012**	0.00	0.18	0.00	2.33	5.85	**0.65**
**2013**	0.00	0.55	0.00	1.75	4.38	**0.66**
**Average (2010-2013)**	**0.08**	**0.30**	**0.32**	**2.62**	**6.48**	**0.82**
**Average (2000-2013)**	**0.06**	**0.30**	**0.67**	**3.32**	**6.40**	**0.89**
**Female**						

Age groups	Age group, years					Overall
	0-17	18-45	46-60	61-75	76+	
**2000**	0.00	0.00	0.00	0.69	0.88	**0.16**
**2001**	0.24	0.14	0.31	2.77	2.45	**0.76**
**2002**	0.00	0.00	0.31	1.39	3.90	**0.54**
**2003**	0.00	0.56	0.93	1.04	5.22	**0.92**
**2004**	0.00	0.28	0.92	1.73	6.57	**1.04**
**Average (2000-2004)**	**0.05**	**0.20**	**0.49**	**1.52**	**3.80**	**0.68**
**2005**	0.28	0.00	0.90	2.80	2.88	**0.88**
**2006**	0.00	0.29	0.00	1.77	2.10	**0.55**
**2007**	0.00	0.43	0.86	1.43	3.40	**0.83**
**2008**	0.00	0.29	0.56	1.82	5.95	**1.00**
**2009**	0.00	0.15	1.38	1.47	1.94	**0.73**
**Average (2005-2009)**	**0.06**	**0.23**	**0.74**	**1.86**	**3.25**	**0.80**
**2010**	0.00	0.00	0.86	1.11	2.50	**0.57**
**2011**	0.00	0.35	0.87	1.86	2.38	**0.86**
**2012**	0.00	0.00	0.29	1.49	2.91	**0.62**
**2013**	0.00	0.18	0.00	0.37	1.18	**0.25**
**Average (2010-2013)**	**0.00**	**0.13**	**0.51**	**1.21**	**2.24**	**0.58**
**Average (2000-2013)**	**0.04**	**0.19**	**0.58**	**1.55**	**3.16**	**0.69**

Table S6. Mortality ASR (W) of Chronic Myeloid Leukemia (per 100 000 per year).

Overall	All population	Males	Females
**2000**	0.13	0.22	0.08
**2001**	0.55	0.74	0.44
**2002**	0.40	0.61	0.25
**2003**	0.64	0.76	0.59
**2004**	0.64	0.77	0.55
**Average (2000-2004)**	**0.47**	**0.62**	**0.38**
**2005**	0.53	0.57	0.52
**2006**	0.40	0.51	0.31
**2007**	0.72	0.98	0.48
**2008**	0.60	0.68	0.54
**2009**	0.35	0.29	0.43
**Average (2005-2009)**	**0.52**	**0.61**	**0.46**
**2010**	0.51	0.82	0.29
**2011**	0.42	0.40	0.46
**2012**	0.32	0.40	0.26
**2013**	0.28	0.47	0.13
**Average (2010-2013)**	**0.38**	**0.52**	**0.29**
**Overall**	**0.46**	**0.59**	**0.38**

Table S7. Relative Survival Rate of Chronic Myeloid Leukemia during 2000-2013 according to age group (3 age groups).

**Population at 2000-2013 period**									
**Age group, years**	**1 year RS**								
	**All population**			**Males**			**Females**		
	**RS**	**95% CI**		**RS**	**95% CI**		**RS**	**95% CI**	
0-17	0.80	0.41	0.95	0.67	0.19	0.91	1.00	-	-
18-60	0.91	0.87	0.94	0.90	0.84	0.94	0.92	0.86	0.96
61+	0.56	0.50	0.61	0.54	0.45	0.61	0.58	0.49	0.65
**Overall**	**0.72**	**0.68**	**0.76**	**0.71**	**0.65**	**0.76**	**0.74**	**0.68**	**0.79**
**5 year RS**									
0-17	0.48	0.16	0.75	0.48	0.09	0.80	0.50	0.06	0.85
18-60	0.73	0.67	0.79	0.74	0.65	0.82	0.73	0.63	0.80
61+	0.27	0.21	0.34	0.29	0.21	0.39	0.26	0.18	0.35
**Overall**	**0.49**	**0.45**	**0.54**	**0.51**	**0.44**	**0.57**	**0.48**	**0.41**	**0.55**
**10 year RS**									
0-17	-	-	-	-	-	-	-	-	-
18-60	0.58	0.49	0.66	0.60	0.47	0.71	0.56	0.44	0.68
61+	0.14	0.07	0.23	0.07	0.01	0.26	0.17	0.09	0.27
**Overall**	**0.36**	**0.31**	**0.42**	**0.37**	**0.28**	**0.46**	**0.36**	**0.28**	**0.44**
**Population at 2000-2004 period**									
**Age groups**	**1 year RS**								
	**All population**			**Males**			**Females**		
	**RS**	**95% CI**		**RS**	**95% CI**		**RS**	**95% CI**	
0-17 years	1.00	-	-	-	-	-	1.00	-	-
18-60 years	0.88	0.80	0.93	0.86	0.74	0.93	0.90	0.77	0.96
61+ years	0.40	0.32	0.48	0.35	0.24	0.46	0.46	0.34	0.57
**Overall**	**0.61**	**0.55**	**0.67**	**0.58**	**0.49**	**0.66**	**0.65**	**0.55**	**0.73**
	**5 year RS**								
0-17 years	-	-	-	-	-	-	-	-	-
18-60 years	0.58	0.47	0.67	0.55	0.41	0.68	0.60	0.45	0.73
61+ years	0.14	0.09	0.22	0.12	0.05	0.23	0.17	0.09	0.28
**Overall**	**0.33**	**0.27**	**0.40**	**0.32**	**0.24**	**0.41**	**0.35**	**0.26**	**0.44**
**Population at 2005-2009 period**									
**Age groups**	**1 year RS**								
	**All population**			**Males**			**Females**		
	**RS**	**95% CI**		**RS**	**95% CI**		**RS**	**95% CI**	
0-17 years	0.60	0.13	0.88	0.50	0.06	0.85	1.00	-	-
18-60 years	0.92	0.84	0.96	0.91	0.78	0.97	0.94	0.81	0.98
61+ years	0.71	0.61	0.80	0.71	0.56	0.83	0.72	0.57	0.82
**Overall**	**0.81**	**0.74**	**0.86**	**0.79**	**0.70**	**0.87**	**0.82**	**0.73**	**0.89**
	**5 year RS**								
0-17 years	0.40	0.05	0.76	0.25	0.01	0.67	1.00	-	-
18-60 years	0.81	0.71	0.88	0.84	0.69	0.93	0.78	0.62	0.88
61+ years	0.37	0.26	0.48	0.40	0.25	0.57	0.34	0.21	0.48
**Overall**	**0.58**	**0.50**	**0.65**	**0.61**	**0.49**	**0.71**	**0.55**	**0.44**	**0.65**
**Population at 2010-2013 period**									
**Age groups**	**1 year RS**								
	**All population**			**Males**			**Females**		
	**RS**	**95% CI**		**RS**	**95% CI**		**RS**	**95% CI**	
0-17 years	1.00	-	-	1.00	-	-	1.00	-	-
18-60 years	0.95	0.86	0.98	0.98	0.81	1.00	0.92	0.77	0.98

61+ years	0.65	0.50	0.77	0.69	0.48	0.84	0.60	0.36	0.78
**Overall**	**0.83**	**0.74**	**0.89**	**0.85**	**0.72**	**0.93**	**0.80**	**0.67**	**0.89**
	**5 year RS**								
0-17 years	-	-	-	-	-	-	-	-	-
18-60 years	-	-	-	-	-	-	-	-	-
61+ years	-	-	-	-	-	-	-	-	-
**Overall**	**-**	**-**	**-**	**-**	**-**	**-**	**-**	**-**	**-**

Table S8. Kaplan–Meier analysis of Chronic Myeloid Leukemia.

**All patients**				
Period	No. of cases	Median survival	95% CI for median survival	
			Lower Bound	Upper Bound
2000-2004	263	21	14	28
2005-2009	212	60	32	88
2010-2013	126	N/R	N/R	N/R
**0-17 year old patients**				
Period	No. of cases	Median survival	95% CI for median survival	
			Lower Bound	Upper Bound
2000-2004	2	16	4	28
2005-2009	5	17	2	32
2010-2013	3	N/R	N/R	N/R
**18-60 year old patients**				
Period	No. of cases	Median survival	95% CI for median survival	
			Lower Bound	Upper Bound
2000-2004	107	69	46	92
2005-2009	91	N/R	N/R	N/R
2010-2013	66	N/R	N/R	N/R
**Over 60 year old patients**				
Period	No. of cases	Median survival	95% CI for median survival	
			Lower Bound	Upper Bound
2000-2004	154	7	4	10
2005-2009	116	28	16	40
2010-2013	57	29	N/R	N/R

Table S9. ECOG performance score of CML patients presenting during 2010-2013 period

**Age**	**ECOG**					
	**0**	**1**	**2**	**3**	**4**	**Total**
0-44	19 (63%)	11 (37%)	0 (0%)	0 (0%)	0 (0%)	**30 (33%)**
45-54	10 (67%)	2 (13%)	1 (7%)	2 (13%)	0 (0%)	**15 (16%)**
55-64	13 (54%)	8 (33%)	2 (8%)	1 (4%)	0 (0%)	**24 (26%)**
65-74	4 (29%)	9 (64%)	1 (7%)	0 (0%)	0 (0%)	**14 (15%)**
75+	1 (11%)	1 (11%)	2 (22%)	3 (33%)	2 (22%)	**9 (10%)**
**Total**	**47 (51%)**	**31 (34%)**	**6 (7%)**	**6 (7%)**	**2 (2%)**	**92 (100%)**

Figure S1. Kaplan–Meier diagram of Chronic Myeloid Leukemia: All age groups.

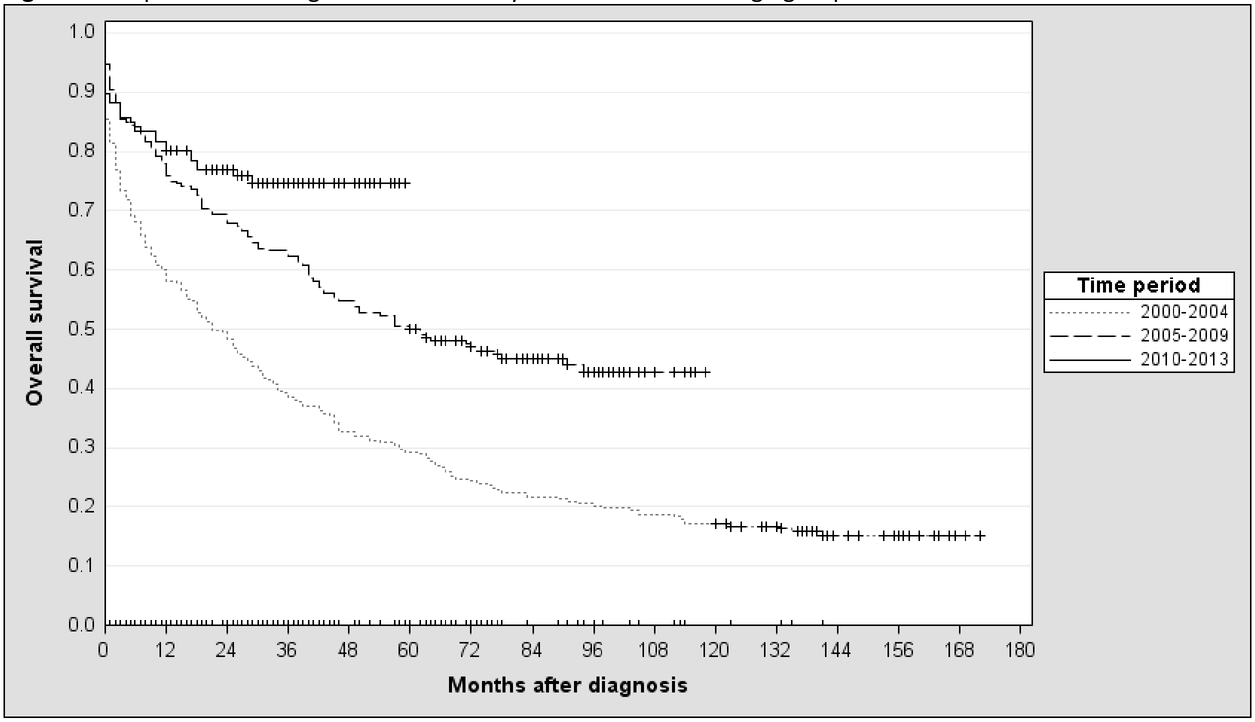

The overall survival comparison (log rank test):

- p < 0.001: 2000-2004 vs. 2005-2009
- p < 0.001: 2000-2004 vs. 2010-2013
- p = 0.957: 2005-2009 vs. 2010-2013

Figure S2. Kaplan–Meier diagram of Chronic Myeloid Leukemia: 18-60 year old patients.

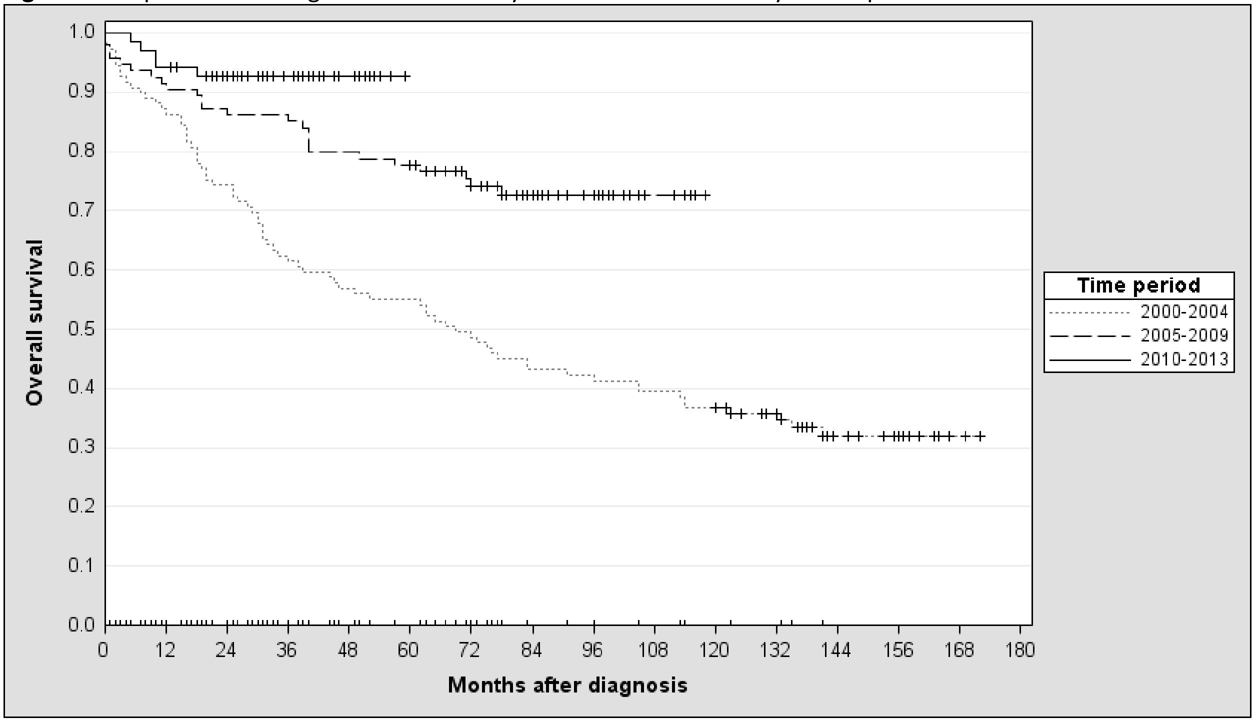

The overall survival comparison (log rank test):

- p < 0.001: 2000-2004 vs. 2005-2009
- p < 0.001: 2000-2004 vs. 2010-2013
- p = 0.755: 2005-2009 vs. 2010-2013

Figure S3. Kaplan–Meier diagram of Chronic Myeloid Leukemia: >60 year old patients.

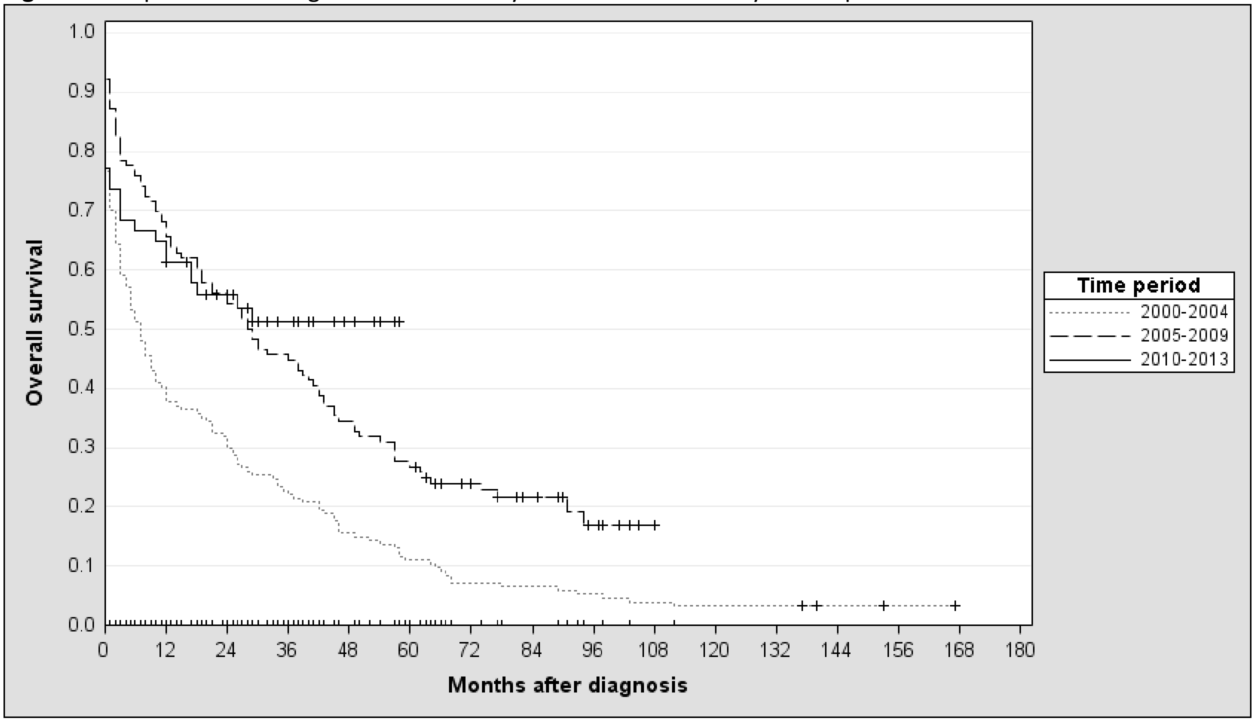

The overall survival comparison (log rank test):

- $p < 0.001$: 2000-2004 vs. 2005-2009
- $p < 0.001$: 2000-2004 vs. 2010-2013
- $p = 0.308$: 2005-2009 vs. 2010-2013

References

1. Dawson, B. and R.G. Trapp, *Basic & clinical biostatistics*. 2001, Monterey: McGraw-Hill.
2. Boniol, M. and M. Heanue, *Age-standardisation and denominators*, in *Cancer incidence in five continents*, M.P. Curado and e. al., Editors. 2008. p. 1-837.
3. *United Nations. World Population Prospects, the 2010 Revision*. 2010; Available from: http://esa.un.org/wpp/Excel-Data/population.htm.
4. Dickman, P.W., et al., *Regression models for relative survival.* Stat Med, 2004. **23**(1): p. 51-64.
